# New perspectives on peer support in an online intervention for family carers of people living with dementia—evidence from an Irish NGO

**DOI:** 10.3389/frdem.2026.1743166

**Published:** 2026-02-20

**Authors:** Fergus Timmons, Enda Donlon, Peter Tiernan

**Affiliations:** 1 Learning and Development Department, The Alzheimer Society of Ireland, Dublin, Ireland; 2 School of STEM Education, Innovation and Global Studies, Dublin City University, Dublin, Ireland

**Keywords:** care partner support, dementia care, family carers, innovative services, Moodle, online learning, peer support

## Abstract

**Introduction:**

The rising prevalence of dementia globally and in Ireland has intensified the need for effective support for family carers, who provide the majority of care for people living with dementia. This study examines an established education intervention called Home Based Care–Home Based Education (HBC–HBE), an online course delivered by The Alzheimer Society of Ireland (ASI). It explores if and how participants found the course to be supportive and investigates the role and importance of peer support in this regard.

**Methods:**

Using a mixed-methods case study approach, the research draws on survey data (*n* = 225) and interviews (*n* = 12). Quantitative data were analyzed using descriptive statistics, while qualitative data underwent template analysis.

**Results:**

Findings indicate that overall participants found the course to be supportive. Research participants reported that peer support on the course helps reduce isolation, enhances confidence, and facilitates knowledge sharing. However, challenges were also identified, including those related to emotional readiness, group dynamics, and technology barriers.

**Discussion:**

The study finds that online peer‑supported education constitutes a valuable source of support for family carers of people living with dementia, notwithstanding certain challenges that also arise. Recommendations are offered in relation to improved course design and structure, including Moodle course usability, developing tutor facilitation skills, and introducing pre-course screening of candidates. Finally, implications in relation to national policies on dementia and digital skills are discussed.

## 1 Introduction

Dementia is a progressive neurocognitive disorder characterized by deterioration in cognitive functions such as memory, reasoning, and language, often accompanied by changes in personality and behaviour (World Health Organisation, 2017). Globally, dementia affects approximately 55 million people, with nearly 10 million new cases annually, and projections indicate that this figure will rise to 78 million by 2030 and 132 million by 2050 (World Health Organisation, 2021). In Ireland, current estimates suggest that 64,000 individuals live with dementia, a number expected to increase significantly in the coming decade (Pierce et al., 2014; Alzheimer Europe, 2020).

The economic and social implications of dementia are profound. Worldwide, direct health care spending on Alzheimer’s disease and related dementias (ADRD) will reach $1.6 trillion by 2050, while the estimated cost of informal dementia care will reach $0.9 trillion by 2050 (Lastuka et al., 2024). The Lancet calculates the macroeconomic burden of Alzheimer’s disease and other dementias (ADODs) to be INT$ 47,479 million in Ireland between 2020 and 2050 which represents 0.401% of GDP between these years (Chen et al., 2024). These are substantial costs and show the value of the role played by family carers in Ireland.

Caring for a person with dementia is associated with significant psychological, physical, and financial strain. Brites et al. (2025) outline how research shows that informal carers of people living with dementia experience high levels of burden, depression, anxiety, distress, higher physical morbidity, social isolation, physical ill health, financial hardship and poor quality of life. Family carers of people living with dementia also report feelings of guilt in relation to their caregiving (Gallego-Alberto et al., 2022). While caregiving can offer emotional rewards and a sense of purpose (Lloyd et al., 2016), the unpredictable trajectory of dementia, coupled with behavioral and personality changes, often exacerbates carer distress (Chiao et al., 2015).

In Ireland, the *Irish National Dementia Strategy* (Department of Health, 2014) emphasizes the importance of enabling people with dementia to live well in their communities for as long as possible, supported by family carers who are confident and competent in their role. Education and training are central to achieving this goal, to equip carers with knowledge, practical skills, and emotional resilience. A more recent strategy document—*The Model of Care for Dementia in Ireland*—again emphasizes the centrality of support for family carers stating ‘100% of supporters/family carers should be informed about and offered education and skills training’ (Begley et al., 2023, p. 15). A recent research report by The Alzheimer Society of Ireland which captured the views and lived experience of over 500 family carers again highlights the need ‘for education, training and advice’ (The Alzheimer Society of Ireland, 2023, p. 38).

The past decade has witnessed a surge in online interventions (sometimes called eHealth interventions) aimed at supporting dementia family carers. Interventions have been widely reported on in the literature, and have taken place worldwide in places such as the United States (Kajiyama et al., 2013; Davis et al., 2014; Pagán-Ortiz et al., 2014), France (Cristancho-Lacroix et al., 2015), Netherlands (Blom et al., 2015; Mierlo et al., 2015; Boots et al., 2016, 2018; Hattink et al., 2016). These interventions potentially offer flexibility, accessibility, and scalability, particularly relevant in contexts where face-to-face training is impractical or unavailable, such as during the COVID-19 pandemic (Bozkurt and Zawacki-Richter, 2021). Systematic reviews which are reviews of existing research ‘using explicit, accountable rigorous research methods’ (Gough et al., 2017, p. 5) suggest that online programs can improve carers’ knowledge, reduce stress, and enhance coping strategies (Boots et al., 2014; Klimova et al., 2019; Frias et al., 2020). However, findings in some systematic reviews are mixed, with some studies reporting modest or non-significant effects on psychological outcomes (Sherifali et al., 2018; Leng et al., 2020).

A recurring theme in the literature is the importance of peer support within online learning environments. A systematic review from 2021 of online peer support found that in 11 out of 15 interventions that had a psychosocial and educational element, ‘statistically significant positive changes were recorded for caregiver knowledge, mental health, stress, depression, distress, burden, self-efficacy, mastery, helplessness and perceived support (while) qualitative outcomes included perceived reductions in stress and increased emotional and informational support’ (Wallace et al., 2021, p. 1).

Peer support is defined within the healthcare context as ‘….the provision of emotional, appraisal, and informational assistance by a created social network member who possesses experiential knowledge of a specific behaviour or stressor and similar characteristics as the target population, to address a health-related issue of a potentially or actually stressed focal person’ (Dennis, 2003, p. 329).

This paper focuses on if and how peer support is achieved (or not) through peer interactions on an online education and training course for family carers of people living with dementia. Previous studies of similar interventions have shown that peer interaction—through discussion forums, video meetings, or social networking features—can mitigate isolation, foster emotional support, and facilitate the exchange of practical caregiving strategies (Hopwood et al., 2018). Moreover, multi-component interventions that combine informational content, professional guidance, and peer interaction are associated with the most positive outcomes (Boots et al., 2014; Etxeberria et al., 2021).

The literature suggests that peer support may offer unique benefits in dementia care education. It can enable carers to share experiences, validate feelings, and learn from others facing similar challenges. This sense of solidarity can enhance confidence and reduce feelings of isolation (Whitlatch and Orsulic-Jeras, 2017). However, peer interaction is not universally beneficial. Group dynamics, emotional readiness, and heterogeneity of caregiving contexts can limit the effectiveness of peer support. For example, spousal carers may have different needs and emotional responses compared to secondary carers, leading to mismatched expectations within group discussions (Teahan et al., 2021). Additionally, some carers may feel overwhelmed by sharing personal experiences or uncomfortable discussing sensitive topics in group settings (Cristancho-Lacroix et al., 2015).

Technological barriers can further complicate peer interaction. While synchronous video meetings can foster real-time engagement, asynchronous forums are sometimes perceived as impersonal or time-consuming (Chiu and Eysenbach, 2011). Usability issues, such as complex navigation or information overload, can deter participation, particularly among carers with limited digital literacy (Hattink et al., 2016).

Despite growing evidence on the role and efficacy of peer support in online interventions, there is limited research exploring its impact within structured, tutor-facilitated courses for dementia family carers in Ireland. Existing studies often focus on pilot programmes or short-term interventions, leaving questions about sustained engagement and practical implementation unanswered (Christie et al., 2018). Moreover, few studies examine peer support from the perspective of carers themselves, beyond quantitative measures of stress or burden (McKechnie et al., 2014).

This study addresses these gaps by investigating if and how an established online education intervention Home Based Care–Home Based Education (HBC–HBE) a course delivered by The Alzheimer Society of Ireland since 2016 supports family carers of people living with dementia. It also examines the role and importance of peer support in this context.

The HBC–HBE course was developed by The Alzheimer Society of Ireland (ASI) in collaboration with European partners under Erasmus+ funding (Timmons, 2018). The course lasts 7 weeks and is delivered fully online via ASI’s Moodle Workplace Learning Management System. A Learning Management System ‘provides teachers with a means to create and deliver content, monitor student participation and assess student performance. It may also allow students to use interactive features such as threaded discussions, video conferencing and discussion forums’ (Cheng and Yuen, 2018, p. 241). In what follows, the terms ‘course participants’ and ‘learners’ are used interchangeably depending on the context of the sentence to refer to people who have completed HBC–HBE and/or who have participated in this research.

The course includes:

E-books and factsheets covering dementia care topics. E-books are also available as audiobooks/podcasts for ease of use. They contain text, graphics and embedded videos. Factsheets cover and re-emphasize key topics in dementia care.

Tutor-moderated discussion forums for asynchronous peer interaction. Each week course participants can interact with each other and their tutor. In the first week of the course all course participants are invited to introduce themselves on a course wide discussion forum. From week two onwards a series of pre-set questions are set out in these forums that encourage sharing of experiences around particular dementia care topics. The tutor moderates these forums and provides feedback and validation of posts made by course participants.

Weekly synchronous video meetings using BigBlueButton Moodle Workplace’s integrated video conferencing software. These meetings last 1 h and consist of a group of up to 20 course participants, one tutor and one technical support staff member. On the first evening of the course, participants are invited to introduce themselves and to share a little about their care situation. In the following weeks, the tutor will summarize each topic—typically there are two topics per week—and then ask the group if they have any experiences they want to share in relation to the topic. These discussions are facilitated in an empathetic, non-judgmental way and aim to include as many experiences and perspectives as possible.

Reflective assignments with personalized tutor feedback. The reflective assignment which are called Weekly Reviews encourage participants to apply what they have learned on the course to their own particular care setting. They are private and are submitted to tutors who also provide private feedback directly to the course participants. It is hoped that these reflective assignments help course participants get expert, tailored advice on particular challenges that they may have faced or find particularly challenging.

Private Messaging between course participants via the Message feature on Moodle. This is completely private and course tutors do not see these interactions. These interactions are only visible to people who send or receive messages.

The pedagogical approach is grounded in social constructivism (Vygotsky, 1978), promoting collaborative learning and peer engagement. It is hoped that this interactivity will promote ‘levels of understanding and performance that potentially exceed independent learning’ (Salvador, 2019, p. 333). Tutors on the course seek to encourage information sharing among course participants, but in a gentle and empathetic manner. A key element of HBC–HBE is the promotion of a person-centered care approach to support course participants informed by the work of Kitwood (1997). Course participants continue to have access to course materials via the Moodle Workplace platform for 5 years from their last log in. This is to allow them to engage with materials on an ongoing basis as they continue their dementia care journey and as the symptoms of the person they are caring for change and develop over time.

All learning activities are voluntary and there are no assessments. Learners are free to decide if and how, and to what extent they engage in the various learning activities. Tutors have a variety of backgrounds including nursing or psychiatric nursing, social care workers, dementia advisers, or Care Centre managers. Some are former family carers and therefore have direct experience of the realities and challenges faced by course participants.

During live synchronous video meetings, the lead tutor is joined by a second tutor/technical support. The role of technical support is to try to make sure each learner can participate in the meetings—usually this means helping with any audio or video issues. In addition, there is technical support available to learners via a dedicated email address and phone number. The course provider has also provided a range of ‘how to’ videos on the HBC–HBE course page. A short biography and contact details of all tutors and support staff is listed in the ‘Introduction to the Course’ book available to learners in week one of the course.

The course is delivered over 7 weeks, with the following topics: Week 1: Welcome and Induction; Week 2 Introduction to dementia, looking after yourself; Week 3 Changing Relationships and Accessing Information; Week 4 Communication and Staying Active; Week 5 Responsive Behaviours and Safety at Home; Week 6 Personal Care and Nutrition and Eating Well; Week 7 Course Review and Next Steps.

The course was first piloted in 2016 with 57 course participants. Since then, it has grown in popularity and uptake, with a gradual increase in annual numbers of participants to 89 by 2019. When the COVID pandemic arrived in 2020, all in-person training ceased and all ASI education and training moved online. Consequently, 391 people participated in HBC–HBE in 2020. These numbers remained high even after the end of the pandemic. In 2024 almost 500 people completed HBC–HBE. The course is delivered five times each year, with 120–140 participants on each course.

## 2 Materials and methods

### 2.1 Research aims

This paper has two aims (1) to show if and how HBC–HBE an online course supports family carers of people living with dementia, and (2) to examine the role and importance of peer support in facilitating such support. By focusing on carers’ lived experiences, this research aims to contribute to the development of more effective, user-centered online education programs and inform policy initiatives aimed at supporting family carers in Ireland and beyond.

### 2.2 Research design

This study employed an intrinsic case study methodology (Stake, 1995; Yin, 2014). Yin defines a case study as ‘an empirical inquiry that investigates a contemporary phenomenon (the “case”) in depth and within its real-world context, especially when the boundaries between phenomenon and context may not be clearly evident’ (Yin, 2014, p. 16).

The research used multiple methods to investigate the experiences of family carers of people living with dementia on an online course. Part A of this research consisted of an online questionnaire. The online questionnaire contained 32 questions, of which 25 were closed and seven were open-ended. The questionnaire was distributed via email in November 2021 and remained open for 2 weeks. The online questionnaire included questions regarding participants’ perceptions of the course’s supportive nature and examined the role of peer support in fostering feelings of support among family carers of individuals living with dementia.

Part B of this research consisted of one-to-one interviews. Interview questions were developed following analysis of responses to the online questionnaire. The interviews were semi-structured. The interviews investigated participants’ perceptions of the course as a source of support and examined the mechanisms through which peer support was manifested. The issue of emotional readiness was raised in the first interview, and subsequent interviewees were asked to comment on their feelings around this topic.

An interpretivist paradigm guided the research. This emphasized the subjective experiences of family carers as fellow learners engaging with each other in one component of what Ball and Forzani (2007) call the ‘instructional dynamic’ (the others being engagement with course content, tutors and the learning environment).

### 2.3 Ethical approval

Ethical approval was obtained from Dublin City University REC (DCUREC/2021/139). Informed consent was secured for both questionnaire and interviews. A participant support plan was implemented to manage potential emotional distress during interviews.

### 2.4 Participants and data collection

A convenience sampling strategy was used for both Part A and Part B of this research. Invitations to complete Part A the online questionnaire of this research were sent to 697 course participants who completed HBC–HBE between 2019 and 2021. Of these 303 accessed the online questionnaire, 225 completed demographic questions. In Part B of the research 12 people participated in semi-structured interviews.

The online questionnaire was distributed via Qualtrics software. It comprised 32 questions (25 closed, 7 open-ended) addressing course components, peer support, and perceived impact. All responses were anonymous. A note was placed at the end of the online questionnaire inviting those interested in being interviewed to email the lead researcher.

The semi-structured interviews were conducted via Zoom (average duration: 52 min). They were transcribed verbatim by the researcher and pseudonyms are used below in the Results section. Interviews focused on experiences of peer support, emotional readiness, and suggestions for improvement. Research participants were required to read, agree and sign consent forms for Parts A and B of the research. Their anonymity was guaranteed for Part A the online questionnaire. In Part B, each participant was given a pseudonym to protect their anonymity.

### 2.5 Data analysis

Closed questions on the online questionnaire were analyzed quantitatively using simple descriptive statistics via SPSS. Interviews and open ended questions in the online questionnaire were imported into NVivo and then analyzed using a particular form of thematic analysis called Template Analysis (Brooks et al., 2015; King and Brooks, 2018). Template analysis is a type of ‘Codebook’ analysis (Braun and Clarke, 2021) that allows for the development of a thematic template, which was developed and refined throughout the research phases.

The initial template was informed by literature-derived themes (information, education, peer/tutor support, learning environment, psychological support). Thereafter, an iterative refinement produced final thematic structure aligned with research questions. All qualitative data was coded using ‘descriptive’ and ‘values’ coding (Saldaña, 2021).

## 3 Results

### 3.1 Demographic information

Respondents to the online questionnaire were mostly female (84.89% or 191 people). The remainder were male (13.33% or 30) or preferred not to say (1.33% or 3). One person described themselves as non-binary/third gender (0.44%). Most respondents were experienced computer users, describing themselves as moderately (43%) or extremely (41%) confident using computers and IT equipment. Nobody described themselves as ‘not at all confident’, though 3% described themselves as ‘slightly confident’ and 13% as ‘somewhat confident’. Overall, 31 people (14% of total) completed the course in 2019, 106 (47%) in 2020 and 87 (39%) in 2021. Primary carers made up 47% of respondents, with secondary carers comprising 53% of respondents. Primary carers provide ‘the most care as opposed to secondary carers (carers who provide only some of the care and are ancillary to the primary carer)’ (Greenwood et al., 2009, p. 339).

Regarding the interview candidates 10 were female and two were male. Two completed the course in 2019, four in 2020 and six in 2021. Six Interviewees were secondary carers, five were primary or joint primary carers, and one was both a primary and secondary carer.

### 3.2 HBC–HBE: to what extent did it support course participants

**Figure 1:**
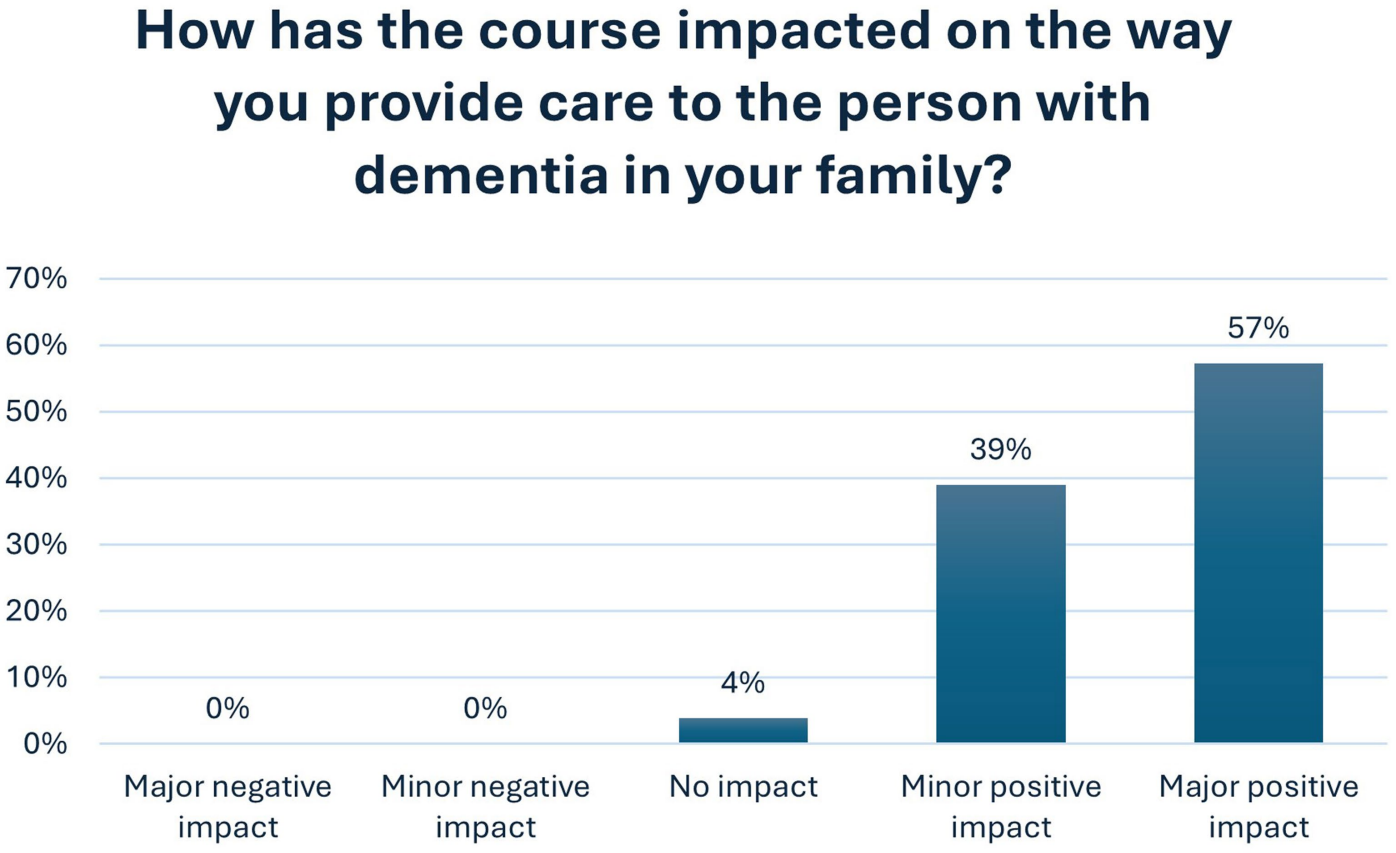
Positive impact of HBC–HBE on course participants.

Overall, 96% of respondents felt that participating in the course had a positive impact on ‘the way you provide care directly or indirectly to the person with dementia’—39% reported a minor positive impact, while 57% reported a major positive impact. No respondents reported a negative impact, while 4% reported no impact (Q. 28 online questionnaire). The full set of figures are provided in Figure 1.

Respondents were also asked about how the course supported them in their care situation (Q.27 of the online questionnaire). A large number (84%) agreed or strongly agreed that the course provided useful ideas on maintaining an active lifestyle for the person to whom they were providing care. The same figure (84%) also agreed or strongly agreed that the course provided useful ideas about the provision of personal care. A slightly lower, but still significant 70% of respondents agreed or strongly agreed that the course provided new ideas about maintaining an active lifestyle for themselves. However, 91% of respondents agreed or strongly agreed that the course gave them helpful ideas about how to maintain a safe care environment.

Adoption of person-centered care principles was also evident. Jessica explained:

*“…what I have learned is that it’s not black or white it’s actually grey, and blue…. you have to look at the holistic approach… you have to frame your message very much around what’s going on for him at that time… my father is very much; he’s still a person.”* (Interview: Jessica, primary carer).

**Figure 2:**
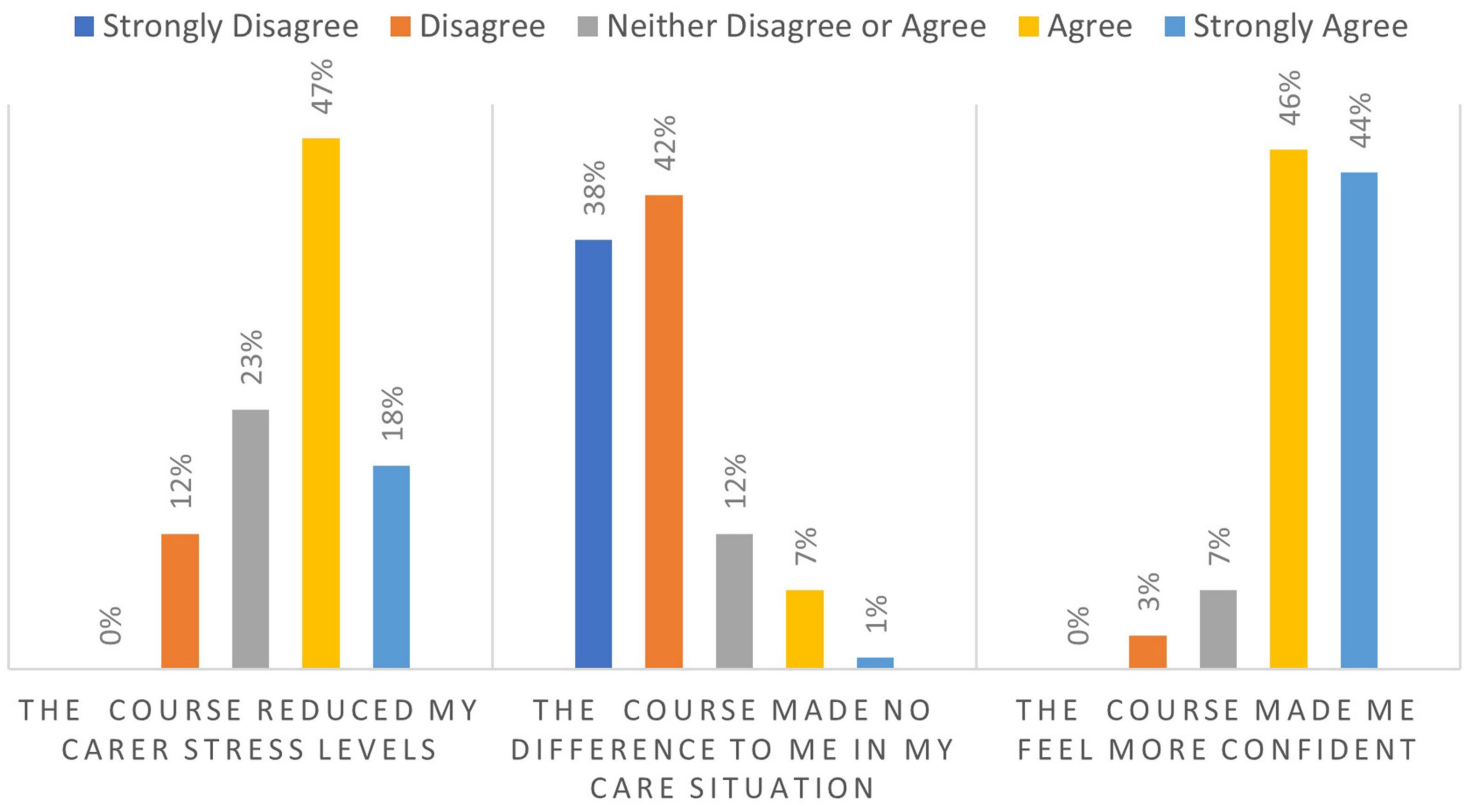
Impact of HBC–HBE on learners’ emotions.

In addition, 65% of respondents to the online questionnaire agreed or strongly agreed that participating in the course reduced their carer stress levels. The vast majority of respondents disagreed that the course made no difference to their care situation. A strong majority of 90% agreed or strongly agreed that the course made them feel more confident. See Figure 2 for more details:

The majority of learners reported high satisfaction levels with their participation in the course, with 89 positive comments recorded. Respondents frequently used descriptors such as *“brilliant”* (Online Questionnaire Respondent (OQR): 38), *“extremely beneficial”* (OQR: 63), and *“excellent course”* (OQR: 182). One participant summarized the transformative impact:

*“The HBC–HBE course has enhanced how I manage my father who is living with dementia. This in turn has had a direct positive impact on the quality of his relationship with myself, siblings and extended family. I learned so much and received really great practical advice. It’s a great programme and I have recommended it to colleagues.”* (OQR: 96)

Learners valued long-term access to course materials, recognizing the importance of being able to access materials on an ongoing basis as the dementia progresses:

*“In the future, I think the course materials will become more important as dad’s needs change and I know I have the course materials and resources to draw on when that time comes.”* (OQR: 122)

Twenty-four participants reported significant changes in caregiving practices. One learner reflected: *“I found the course extremely beneficial. The space to voice concerns and speak openly without judgement should not be underestimated. It provided a great source of comfort to me, and I could not speak highly enough of it.”* (OQR: 270).

Gratitude toward the course provider and course facilitators was widespread:

*“I’m glad you’re doing this work… it is badly needed and will make big changes to very vulnerable people so fair play to you. I can honestly say these courses should be compulsory if families wish to care for the people in their own homes… education is key to quality care…. Thank you.”* (OQR: 39)

Notably, 36 respondents indicated they had no suggestions for improvement, with comments such as: *“I have nothing to offer to this question… I think the attitude of the course tutors and ethos in the way the course is delivered is just right!”* (OQR: 208).

### 3.3 Suggestions for improvement

Despite overall positive feedback, some learners expressed dissatisfaction with the course. One secondary carer noted:

*“Perhaps it was my situation, I was the secondary carer. And I suppose you could follow up all the information provided and study and think about the information in your own time to get more out of the course. But there was just something about it that didn’t meet my needs, sorry.”* (OQR: 32)

Others criticized online delivery during COVID-19 restrictions: *“I found doing the course online to be of little benefit to me. I know with the current COVID restrictions that it wasn’t possible any other way.”* (OQR: 140).

Learners proposed structural changes, such as adding a mid-course review week: *“It would be very beneficial to add an extra week mid-course… oftentimes it’s not until a week or so after that discussion points/questions come to mind.”* (OQR: 271) Content-related gaps were highlighted, particularly for early-onset dementia: *“The course material seems to cover Dementia in the elderly. The person I care for has early onset… there was very little information to prepare us for that.”* (OQR: 167) Other suggestions included consolidating factsheets into a single PDF, providing bullet-point summaries, and incorporating relaxation exercises or expert input from nutritionists and occupational therapists. Calls for ongoing support were also suggested: *“I think it would be great if on the anniversary of the course all the participants were invited to a video meeting to see how we are getting on and to reconnect.”* (OQR: 22)

Finally, some advocated for specialized courses: *“I would really like to see the Alzheimer’s Ass doing courses for rare dementia… I’ve had to go to UK support groups to get proper information.”* (OQR: 75)

These findings underscore the HBC–HBE course’s strong impact on family carers of people living with dementia. Positive experiences were linked to practical advice, person-centered care and ongoing resource access. However, dissatisfaction highlights the need for flexible delivery, tailored content, and ongoing engagement. Addressing these areas could enhance inclusivity and long-term effectiveness.

### 3.4 Understanding the contribution of peer support in HBC–HBE

As noted above Ball and Forzani (2007) referred to the ‘instructional dynamic’ as the course participants complex interaction with course content, course tutors, fellow learners and the learning environment. What became clear in this research is that all elements of the instructional dynamic contributed to course participants’ feelings that HBC–HBE was mainly supportive to them in their role as family carers of people living with dementia.

Participants on HBC–HBE were asked in the online questionnaire to rate how supportive various elements of the instructional dynamic were for them. Table 1 shows that ‘interactions with your fellow course participants’ was ranked second behind interactions with course tutors, and ahead of reading the course materials and completing weekly reflective assignments. In response to Q.33 of the online questionnaire (How supportive did you find the following learning activities of the course? To what extent did they help you provide care in your dementia situation), 41% of respondents found these interactions very supportive, while 33% found them extremely supportive, with a total mean score of 3.97.

**Table 1:** How supportive did you find the following elements of the course.

Question	Not at all supportive		Slightly supportive		Moderately supportive		Very supportive		Extremely supportive		Total	Mean
Interactions with your tutor	1%	2	1%	3	10%	21	42%	86	45%	93	205	4.29
Interactions with your fellow course participants	2%	5	6%	13	17%	34	41%	85	33%	68	205	3.97
Reading course materials: e-books, factsheets	0%	1	6%	13	23%	48	50%	103	20%	40	205	3.82
Completing the weekly reflective assignment	2%	4	16%	33	24%	49	39%	78	19%	38	202	3.56

In addition, participants on HBC–HBE were asked how they gained support (defined as support/advice from other learners on the course) from their peers on the course (Q.42 on the online questionnaire: How important was Peer Support [support/advice from other learners on the course]in assisting or informing the care you deliver?). Their answers are set out in Table 2. It shows that participants on HBC–HBC gained support mostly via live video meetings and discussion forums, and to a lesser extent through private messaging on the Moodle course page.

**Table 2:** Sources of peer support on HBC–HBE.

Question	Not at all important		Slightly important		Moderately important		Very important		Extremely important		Total	Mean
Interactions via video meetings	3%	5	6%	11	12%	22	44%	82	35%	65	185	4.03
Interactions via discussion forums	4%	8	10%	19	19%	35	35%	65	31%	58	185	3.79
Interactions via message/private chat feature	14%	25	14%	25	28%	49	27%	47	17%	30	176	3.18

Open ended questions in the online questionnaire sought further insight into how peer support manifested itself. For many (though not all) peer interaction was consistently described as a very valuable element of the course.

It provided a range of important influences on course participants. These included emotional support, practical advice, and a sense of belonging. These connections helped carers feel less isolated, more confident, and better prepared for their caregiving roles. The combination of shared learning, emotional reassurance, and mutual contribution created a powerful support network that extended beyond the course itself. These contributions with supporting quotes are grouped below.

#### 3.4.1 Emotional connection and reduced isolation

It is noteworthy that 88% of respondents agreed or strongly agreed that the course provided opportunities to give and receive support from other participants (Q.25). Participants consistently highlighted the importance of feeling connected and less isolated through peer interactions, as this quote shows: *“I was feeling isolated and when I listened to other stories I did not feel so alone.”* (OQR: 74) This sense of community was described as a lifeline, especially for those who had felt alone in their caregiving journey *“Meeting other people on the course was a great support and I learned a lot from them also and didn’t feel so alone.”* (OQR: 44) Being part of a group where everyone understood the challenges helped to create a strong feeling of solidarity and belonging, *“It felt like somebody really understood.”* (OQR: 120).

The course provided a safe space for sharing experiences, offering comfort and reassurance, as illustrated by this quote *“Interacting with other participants was the most important as it’s reassuring to hear others go through the same challenges you face.”* (OQR: 157).

Participants often mentioned that hearing others’ struggles and successes helped normalize their own feelings, *“The discussion groups every week were really helpful. Sometimes you feel you are the only one caring for a loved one and it is overwhelming. Hearing others’ stories and being able to relate to them helped.”* (OQR: 96) This emotional support was not only comforting but also empowering, as it reassured carers that they were not failing in their roles:

*“In a time when I was struggling to accept, understand and needed support in my new role as carer for my mum, the course offered all that and more. The information was invaluable but it was the sense of community that helped the most. I no longer felt alone. I learned from others experiences and felt more able to assume my role as carer.”* (OQR: 16)

Course participants emphasized the reassurance gained from sharing experiences with others in similar situations:

*“I found it most helpful speaking to the other participants. They were experiencing a lot of the same things, but also feelings and emotions as myself. It was nice to speak to someone outside of my immediate family who could relate to my experience.”* (OQR: 91)

Some also felt that it was positive hearing from other dementia family carers about what lies ahead, and this comment is typical of this sentiment: *“Alzheimer’s was completely new to us. We never had any dealings with dementia in our family. The group session was extremely good as it gave us an idea of what to expect but also how to deal with situations.” (OQR: 266)* Another commented that *“Some carers had loved ones further along the stages of dementia and were able to give us tips because they had already gone through the stage our mother is in.” (OQR: 195)*Others commented about strong bonds and friendships that were facilitated by participating in the course. A female course participant who was a secondary carer and whose father was a spousal carer (and participated on the same HBC–HBE course cohort as her) elaborated on how her father benefitted from the course, and how he built important friendships with peers on the course:

*“Hearing about other carers experiences was of huge benefit to my dad, the primary carer. It helped him feel less alone and more supported on this journey. He has formed bonds and friendships with other carers who took the course. He relies hugely on those other carers, in terms of advice and guidance.” (OQR: 195)*

#### 3.4.2 Knowledge sharing and practical tips

Participants valued learning from others’ experiences, *“Exchanging stories and tips with course participants most helpful.”* (OQR: 65) Some participants gained strategies and coping mechanisms, as in this quotation:

“*The most important thing to me was the interaction with other participants, I found it so helpful hearing their stories and it made you feel that there were others who were facing the same problems as you, which is very supportive. I liked hearing the other people’s stories and the problems they were struggling with and the advice the tutors gave.*” (OQR: 148)

Peer discussions facilitated the exchange of practical strategies for managing dementia-related challenges. Examples included distraction techniques, home safety measures, and communication tips: Another participant noted

“*I found it great to talk and discuss with others on how they are coping and relate ideas and suggestions. Talking to others we decided to get the internet, and I have since put a small camera into Mam's just viewing her sitting room, I can now ring her later in the evening as I can see that she's still up. We got a smart speaker and upgraded the TV. We can turn the TV off remotely.*” (OQR: 129)

Another Interviewee, Julia, was grateful to learn about distraction techniques from a fellow course participant. During our interview she relayed how this helped her to re-orientate the person with dementia, to change conversations or repetitive behaviors, *“And the lady that I look after, I use that exact type of distraction technique all the time with her.”* (Interview: Julia, primary and secondary carer). The issue and importance of contrasting color schemes was taken up by Violet during our interview. She found the tips on decorating and *“making skirting boards have contrasting colors”* very useful.

Participants also appreciated the opportunity to help pass on their experience, as in this quote *“It* (the course) *also provided a chance to give some experience tips to other carers.”* (OQR: 52) Sharing their own experiences and tips gave carers a sense of purpose and achievement *“I found myself able to advise others who were in distress and that lifted my morale.”* (OQR: 91).

#### 3.4.3 Confidence and reassurance

As Figure 2 outlines, 90% of respondents reported feeling more confident in their caregiving role after completing the course (Q.30). 82% actively discussed dementia with family and friends as a result of participating in HBC–HBE (Q.25). Respondents linked this increased confidence at least partly to validation of their care practices and reassurance from peers *“It gave me reassurance about the care path I was following, which in turn helped reduce my stress levels.”* (OQR: 84).

For some course participants peer support boosted confidence and validated participants’ caregiving approaches, as this quote shows *“I gained confidence from the course and from speaking to others who were going through the same.”* (OQR: 48) Many reported feeling more capable and less overwhelmed after hearing practical solutions and reassurance from others, *“I was overwhelmed at the start of the course but feel better equipped to care for my Mam.”* (OQR: 50) For other course participants, this confidence translated into better coping strategies and improved well-being, *“I felt I gained more confidence, and I knew I wasn’t alone, and we all had our chance to share our experiences.”* (OQR: 86).

Feelings of reassurance and increased knowledge of dementia led many to express increased confidence levels, as in this quote:

*“I felt more confident in knowing what to look out for in a person with dementia and trying trial and error with different ways of support, to pick the correct course of action to my father’s needs, the course helped me to realize that not everything is perfect, and each person may require a different approach to care.” (OQR: 171)*

Course participants also commented on feelings of increased confidence and empowerment. One learner commented that the course *“gave me more confidence to learn that I was on the right track with how I was looking after my mother and doing a good job.”* (OQR: 197). Another learner felt *“more confident and comfortable caring for my mother.”* (OQR: 146).

Others commented that the course helped them to find out what supports were available and gave them the confidence to look for those services. In her interview Anne outlined how this was helpful to her:

*“The other thing I would say is definitely information about supports that are available. That was key as well. And the confidence to say no, ‘I know that's available or should be’, or whatever, and that was a huge help* (Interview: Anne, secondary carer).

This comment was backed up by Louise who said that the course ‘pointed me in the right direction for where to go to instigate getting extra care ourselves’ (Interview: Louise, primary carer). Moreover, Louise elaborated on how she felt strong enough to act as family spokesperson to external care authorities and with her own siblings to better organize care for her father.

However, challenges remain in relation to peer support, and these are addressed below.

### 3.5 Challenges relating to peer support

#### 3.5.1 Group dynamics

While most participants valued peer interaction, some expressed frustration with group dynamics during video meetings: *“Too many others wanted to discuss their situations, and I did not feel I had the same issues.”* (OQR: 81).

There were occasions when dominant voices occasionally overshadowed quieter participants, limiting opportunities for balanced discussion. Suggestions for improvement included using smaller breakout groups and subgrouping participants by caregiving role: *“I would prefer people who were in my situation… if you had people who are caring for a parent, because that’s obviously different for somebody caring for a spouse.”* (Interview: Emma, secondary carer).

#### 3.5.2 Emotional readiness

It should be noted that positive emotional support was not uniformly experienced by family carers who had completed HBC–HBE. Emotional readiness in this paper refers to learners not being emotionally ready to participate and learn on the HBC–HBE course. In other words, their emotional state acts as a barrier to more meaningful engagement on HBC–HBE. As one participant noted: *“Although I was engaged in everyone else’s story, I was going through a difficult situation with my siblings regarding my mother’s care. I felt uncomfortable sharing my experiences because of this.”* (OQR: 39).

Emotional readiness is also related to complexities within family relations. For one participant, this included family members’ *“reluctance to acknowledge that a family member has dementia.”* (OQR: 239) One respondent whose family was in *“a very difficult situation’ requested ‘some discussion on future courses’ related to family dynamics.”* (OQR: 239).

For one person this experience of being overwhelmed was because they had just *“received the diagnosis”* and this led that person to *“engage to some extent, but I will go back to the resources as I need them.”* (OQR: 85) Other respondents mentioned shyness (OQRs: 11 and 247), or lack of confidence as reasons for not participating in various aspects of the course, which for one participant meant she was *“afraid I would say the wrong thing.”* (OQR:183).

Others cited fear of upsetting other participants as reasons for low levels of participation in course activities:

*“I did contribute slightly but wasn’t in the headspace to be more involved because I knew my sister was more advanced than the others and I didn’t want to distress the others. It’s quite difficult to establish when we should disclose the level to the other course members.” (OQR: 39)*

This comment shows how caring and compassionate learners are for the welfare of their peers. It also demonstrates the very tricky balance between a learner’s need for support through sharing, and their need to not feel they are negatively impacting on other learners’ feelings and emotions.

#### 3.5.3 Technological barriers

Most respondents found Moodle Workplace easy to use. They found Moodle *“quite easy to access as an online platform,”* (OQR: 158) *“fine, no issues”* (Interview: Anne, secondary carer), or *“efficient, no issues, it was all very clear.”* (Interview: Frances, secondary carer).

However, 20% reported some difficulties in logging in for the first time, 19% had difficulty finding their way around the course page and only 6% had any difficulty participating in video meetings. The full set of figures are outlined in Table 3.

**Table 3:** Learner views on Moodle usability.

Question	Very difficult		Difficult		Neither difficult nor easy		Easy		Very easy		Total	Mean
Logging on for the first time	2%	3	18%	35	23%	45	35%	69	22%	44	196	3.59
Finding my way around the course page	1%	2	18%	36	26%	50	32%	63	23%	45	196	3.58
Participating in video meetings	2%	3	4%	7	12%	23	45%	89	38%	74	196	4.14

Participants mentioned lack of time *and* finding the software clunky to navigate, as in this quotation:

*“I found I had little time available to give to Discussion Forums. I work full time, have a family and was the full-time carer for my mother every weekend. …. I found the software clunky to navigate. It was my first time using such software. But time restrictions were the main reason.” (OQR: 285)*

Another learner found *“the online forums a little intimidating. It took some time to get accustomed to using the Moodle system, and this was a bit distracting at the beginning.”* (OQR:101) Emma explained that she did not participate in the Discussion Forums because she *“found it a bit time consuming even trying to get in to understand them, so I just stuck with the with the [video] meetings.”* (Interview: Emma, secondary carer).

Several HBC–HBE participants made comments related to the usability of Moodle as a learning platform. In her interview Jessica, who had a positive course experience, described Moodle as “*clunky*.” When asked to clarify what she meant by ‘clunky’, she said *“okay, not intuitive, and too many clicks.”* (Interview: Jessica, primary carer).

Others mentioned how a combination of factors impacted their participation. These included issues related to difficulty navigating Moodle, for example *“it took me some time to learn how to navigate the system”* or their own equipment, *“having trouble with my laptop.”* (OQR: 204). Others just mentioned their *“lacking in confidence with navigating around the site”* (OQR: 276) as inhibitors to participation. Another learner found they could not attend the video meetings *“due to their own poor internet quality.”* (OQR: 169).

A couple of learners felt that it was difficult to find information on the Moodle page, as shown in this quote: *“I found there was an awful lot of information [on Moodle, which was] definitely a good thing, but at times I found it extremely overwhelming….and found it took me some time to come to grips with the system.” (OQR: 91).*

Another thoughtful insight into Moodle usability came from this learner who commented:

*“I found Moodle hard to navigate, especially having to scroll through the information to see where I had left off in terms of completed coursework. I also was unsure which questions were private, and which were for the group in the coursework discussions” (OQR: 136)*

## 4 Discussion

### 4.1 Interpretation of findings

This study had two aims (1) to show if and how HBC–HBE an online course supported family carers of people living with dementia, and (2) to examine the role and importance of peer support in facilitating such support. Findings have demonstrated that many participants on HBC–HBE found that the course supported them in their roles as family carers of people living with dementia. Furthermore, it has described *how* these same course participants found the course to be supportive. It details—in many cases through direct quotes from research participants—how the course has had a mainly positive impact on their caregiving practice through improved communication techniques, the acquisition of new care skills and increased confidence in their caregiving role.

The findings of this study confirm that peer support is a critical component of online education for dementia family carers. Participants consistently reported emotional benefits, including reduced isolation and increased confidence, alongside practical gains such as improved communication strategies and care skills. These outcomes align with previous research highlighting the value of multi-component interventions that integrate informational content, professional guidance, and peer interaction (Boots et al., 2014; Hopwood et al., 2018).

Peer support facilitated a sense of solidarity among carers, enabling them to share experiences and validate their feelings. This emotional reassurance is particularly significant given the well-documented psychological burden associated with dementia caregiving (Basu and Mukhopadhyay, 2021; Gilsenan et al., 2023). The opportunity to connect with others who “understand” the challenge of caregiving appears to mitigate feelings of loneliness and foster resilience. Moreover, this sense of confidence and openness empowered family carers to seek out services and supports to which they were entitled.

The positive impact of peer interaction observed in this study echoes findings from interventions such as *Diapason* (Cristancho-Lacroix et al., 2015) and *Partner in Balance* (Boots et al., 2018), where carers valued opportunities for social exchange and emotional support. Similarly, systematic reviews have emphasized that peer support enhances engagement and improves psychological outcomes (Hopwood et al., 2018; Etxeberria et al., 2021).

However, this study also highlights some limitations that mirror concerns in the literature. Emotional readiness emerged as a barrier for some participants, consistent with observations that carers may struggle to confront sensitive topics or share personal experiences in group settings (Chiu and Eysenbach, 2011). In this study a minority of participants reported difficulty engaging due to emotional unpreparedness. For these carers, the emotional intensity of confronting dementia-related realities within a group setting proved challenging.

This is consistent with findings on a study of another online programme for family carers called *Mastery over Dementia*, where ‘The discussion forum was not used because caregivers struggled with shame in the early stages, and sharing their story felt like a betrayal to the care recipient’ (Boots et al., 2018, p. 7). Group heterogeneity—particularly differences between spousal and secondary carers—occasionally diluted the relevance of discussions, reinforcing calls for tailored subgrouping within online interventions (Teahan et al., 2021).

Technology-related challenges reported by some participants, including difficulties navigating Moodle and engaging with discussion forums, align with usability issues documented in studies of other platforms (Boyd et al., 2014; Hattink et al., 2016). While synchronous video meetings were generally well-received, asynchronous forums were perceived as less engaging, echoing findings from *Diapason* where forums were described as “impersonal” (Cristancho-Lacroix et al., 2015).

### 4.2 Practical implications

The findings underscore the need for teaching and learning design strategies that maximize the benefits of peer support (Han et al., 2022; Olesen et al., 2023) while mitigating its limitations. Based on evidence from this study, some recommendations to enhance the effectiveness of peer support include now follow.

Pre-course emotional readiness screening to identify carers who may require additional support before engaging in peer discussions. Psychologists have used the Transtheoretical Model (TTM) to assess the emotional readiness of people to become adoptive parents (Prochaska et al., 2005). Perhaps this tool could be adapted for use as a screening tool for potential HBC–HBE course applicants. It is acknowledged that course organizers would need to be very careful in how they implement this, and pilot testing any such screening process would be advisable.

Further tutor training in facilitation skills to more effectively manage the issue of group dynamics noted above and thus ensure more equitable participation. Tutors have the difficult task of balancing the need to support course participants with empathy and kindness against the need to deliver the education materials in a timely fashion. Feedback suggests that most tutors have found this balance most of the time. But there is undoubtedly a need to constantly review tutor skills and comfort levels as they deliver what can be an emotionally challenging course.

Breakout groups organized by carer type (e.g., spousal vs. secondary) to enhance relevance and empathy. BigBlueButton (the video conferencing software that is integrated into Moodle Workplace) has Break Out Room functionality. Tutors have some knowledge about using Break Out Rooms. More practice should be encouraged to learn how to use it more effectively and flexibly.

Simplification of platform navigation and provision of clear onboarding resources to reduce cognitive load and perceived effort. This would involve the creation of more learner support materials including ‘how to’ support videos, and a learner handbook covering information on course outline, structure, learning resources and activities. In addition, a simplification of onboarding instructions and streamlining of communication methods as participants join the course would be valuable.

Optional asynchronous alternatives for carers with time constraints or limited digital literacy. This could involve the creation of materials that summarize each topic on the course, which would be easy to access from the main HBC–HBE course page.

These measures can enhance the inclusivity of online education programs, ensuring that peer support remains a positive and empowering experience for all participants. The literature suggests that multi-component interventions that include peer support as a vital element are more impactful and helpful to family carers of people living with dementia (Boots et al., 2014; Wallace et al., 2021).

### 4.3 Policy implications

The *Irish National Dementia Strategy* (Department of Health, 2014) and the *Model of Care* (Begley et al., 2023) emphasize the centrality of supporting family carers through education and training. Findings from this study reinforce the importance of embedding peer support within such initiatives. Policymakers should prioritize funding for scalable, user-friendly online programs that combine structured content with interactive peer engagement. This approach aligns with broader health policy goals of sustaining community-based care and reducing reliance on institutional services.

Ireland’s digital literacy policy for adults is primarily articulated in the *Adult Literacy for Life* strategy (Government of Ireland, 2021). This framework addresses adult literacy, numeracy, and digital skills, aiming to reduce the proportion of adults without basic digital skills from 47 to 20%. The strategy emphasizes investment in digital skills provision through both formal and non-formal learning routes. However, the document lacks specific measurable actions, timelines, and accountability mechanisms to achieve these targets.

Complementary reports such as the *Digital Inclusion in Ireland Report* advocate for closing gaps among older adults—a group disproportionately affected by digital exclusion (National Economic and Social Development Office, 2021). Recommended actions include the development of national digital skills learning programs tailored for older people, integration of digital hubs within existing community infrastructure to provide accessible training and ongoing support for digital technology access and usage.

Age Action (2020) further suggests embedding digital literacy initiatives into community networks, ensuring sustainability and reach. Despite these recommendations, current policy remains fragmented and under-resourced, with insufficient funding streams and limited coordination between health, education, and social care sectors.

For dementia carers, this lack of specificity is critical. As we have seen above in Table 3 almost one fifth of research respondents reported some difficulties in using the online learning platform. This may correspond to the 17% of research respondents in Section 3.1 above reporting lower levels of confidence in using computers and IT equipment. Without targeted digital literacy interventions, many older carers cannot fully engage with online training programs like HBC–HBE. Future policy must therefore be specific, adequately funded, and transparent, incorporating measurable targets and cross-sector collaboration.

### 4.4 Study limitations

Several limitations must be acknowledged. The use of convenience sampling may limit the generalizability of findings, and carers with low digital literacy were underrepresented. The quantitative components of the online questionnaire did not use validated outcome measures such as the Zarit scale for caregiver burden. It should be noted that his research did not include a control group for comparator purposes. In addition, there is the possibility of response bias from course participants who completed the online questionnaire in Part A of this research.

The study did not include tutors’ perspectives, which could provide valuable insights into the facilitation of peer support. Additionally, though the research participants had taken part in HBC–HBE over a 3-year period, it nevertheless focused on short-term outcomes; longitudinal studies are needed to assess the sustained impact of peer networks on carer wellbeing.

### 4.5 Future research

Future research can help us further understand the experiences and needs of family carer of people living with dementia, particularly as they relate to online learning initiatives. Some suggestions for future studies include high quality peer-reviewed research on the long-term effects of peer support on psychological resilience and quality of care provision, further qualitative investigation of support to improve our understanding of emotional readiness and its influence on engagement. It would also be useful to further investigate the role of tutor facilitation strategies in optimizing group dynamics, particularly in relation to courses where participants are experiencing stress or burden. Further research could also forensically investigate how digital literacy levels impacted learner activity on online courses for family carers of people living with dementia.

In conclusion, this paper has outlined if and how an online education course has supported family carers of people living with dementia. It has also provided a detailed insight into the role of peer support on HBC–HBE. It is hoped that the areas highlighted above will be of interest to a wide variety of education policy makers and practitioners in the field of education (especially digital and online education). It highlights the importance of trying to create a learning environment that gives people the space and opportunity to express their experiences, emotions and skills to help support their fellow family carers. It has also provided some evidence based ideas on how to further enhance the provision of peer support in online learning.

## Data Availability

The raw data supporting the conclusions of this article will be made available by the authors, without undue reservation.
